# Five Years of HCV Dried Blood Spot Testing in At‐Risk Populations and the Impact of Reduced Sensitivity on Missed Diagnoses

**DOI:** 10.1111/liv.70853

**Published:** 2026-08-28

**Authors:** Nicolas Capelli, Amandine Pisoni, Fadi Meroueh, Anne‐Marie Mondain, Patrick Pastor, Philippe Van de Perre, Ilka Engelmann, Edouard Tuaillon

**Affiliations:** ^1^ INSERM, Pathogenesis and Control of Chronic and Emerging Infections, PCCEI U1058 Montpellier University Montpellier France; ^2^ Laboratory of Virology Montpellier University Hospital Montpellier France; ^3^ Health Unit of the Villeneuve‐Les‐Maguelone Prison Montpellier University Hospital Montpellier France

**Keywords:** community‐based screening, decentralised testing, dried blood spot, hepatitis C elimination, low‐level viremia

## Abstract

**Background & Aims:**

Achieving HCV elimination requires diagnostic strategies capable of reaching underserved populations who remain poorly accessed by conventional healthcare pathways. Dried blood spot (DBS) sampling enables decentralised testing, but concerns remain regarding its analytical sensitivity and the potential risk of missed diagnoses.

**Methods:**

We conducted a 5 year real‐world implementation study of DBS‐based HCV screening in France (2020–2024). DBS samples were collected in harm reduction centers and other community‐based facilities and sent to Montpellier University Hospital for centralised analysis. We evaluated the diagnostic yield of DBS in routine practice and estimated the potential number of missed cases by comparing the DBS detection threshold with the distribution of plasma HCV RNA levels observed at diagnosis in untreated individuals.

**Results:**

The limit of detection (LOD) for HCV RNA on DBS was 1252 IU/mL, with a systematic underestimation compared with plasma measurements (mean bias −2.29 log_10_ IU/mL). Among 1552 individuals tested using DBS, 428 (27.6%) were HCV RNA positive, including five cases with no detectable HCV antibodies. DBS testing accounted for 56.4% of all HCV infections identified in our laboratory during the study period. Based on plasma viral load distributions at diagnosis, 2.7% of infections, corresponding to approximately 12 additional cases, were estimated to be missed due to low HCV RNA levels.

**Conclusion:**

Despite a modest reduction in analytical sensitivity compared with plasma‐based testing, the substantial public health benefits associated with increased testing coverage strongly outweigh this limitation and support the integration of DBS into HCV elimination strategies.

## Key Points

1. DBS‐based HCV testing reached underserved populations in real‐world settings.
2. Analytical sensitivity of DBS is reduced but clinically acceptable.
3. Only ~2% of infections may be missed due to low‐level viremia.
4. Public health benefits of DBS outweigh its lower analytical sensitivity.

**Abbreviations**
CI: confidence intervals
CV: coefficient of variation
DAA: direct‐acting antiviralS
DBS: dried blood spot
EASL: European Association for the Study of the Liver
LOD: limit of detection
LOQ: limit of quantification
PWID: people who inject drugs
SD: standard deviation

## 1 Introduction

France has been identified among the European countries capable of achieving the World Health Organiszation's (WHO) hepatitis C elimination targets by 2030 [1, 2]. The use of direct‐acting antiviral (DAA) therapies has led to a substantial decline in the prevalence of chronic HCV infection, from an estimated 0.8% in 2011 to 0.3% in 2019 [3, 4].

Despite this progress, identifying and engaging individuals who are unaware of their infection remains a critical unmet need. People who inject drugs (PWID) represent one of the populations most affected by HCV infection globally and continue to face substantial barriers to testing, diagnosis and linkage to care. A recent systematic review highlighted that HCV testing uptake among PWID remains suboptimal in many settings worldwide, despite the availability of effective testing strategies. In Europe, PWID account for more than one third of prevalent HCV cases [4, 5]. Addressing the needs of these individuals requires implementing targeted strategies to overcome the structural and social barriers to testing, diagnosis and care.

In this context, dried blood spot (DBS) sampling has emerged as a practical and minimally invasive alternative to conventional venous sampling. Fifteen years ago, we implemented HCV diagnosis using DBS in our hospital [6]. HCV testing based on decentralised blood collection on DBS with centralised laboratory analysis has been demonstrated as an effective intervention to enhance HCV testing in various contexts including among PWID [7, 8, 9, 10, 11, 12]. Meta‐analyses have confirmed that DBS is a reliable tool for serological and molecular screening, with pooled sensitivities and specificities exceeding 95% for anti‐HCV and HCV RNA detection [7]. DBS‐based testing has been incorporated into WHO, EASL and French national guidelines as a recommended strategy to enhance access to HCV diagnosis, particularly among hard‐to‐reach populations [13, 14, 15].

Although DBS‐based molecular testing offers substantial operational advantages, its slightly reduced analytical sensitivity compared to conventional plasma assays may result in missed diagnoses in individuals with low‐level viremia [7]. This highlights the importance of assessing the real‐world clinical impact of this limitation under operational conditions, especially in the context of population‐based screening strategies. The present study evaluates DBS‐based HCV testing between 2020 and 2024 across a large network of addiction treatment centres. We report the number of individuals screened and diagnosed, and estimate the proportion of potentially missed infections due to low‐level viremia falling below the detection threshold of the current DBS method.

## 2 Materials and Methods

### 2.1 Study Setting and Design

Our laboratory at Montpellier University Hospital routinely performs DBS‐based HCV testing on samples collected in specialised facilities providing counselling, medical and psychosocial support to people who inject drugs and other vulnerable populations. Between January 2020 and December 2024, DBS samples were collected in harm reduction centers and other community‐based facilities and sent to Montpellier University Hospital for centralised analysis. No written consent has been obtained from the patients as no patient‐identifiable data included. The primary objective was to estimate the proportion of potentially missed diagnoses attributable to reduced analytical sensitivity. HCV RNA testing on DBS was performed in all individuals who tested positive for anti‐HCV antibodies. In individuals who tested negative for anti‐HCV antibodies, molecular testing was performed only upon specific request from the prescribing screening centres, based on clinical assessment of recent HCV exposure risk. As a secondary objective, we evaluated the frequency of HCV RNA positivity among individuals who tested negative for anti‐HCV antibodies on DBS. Samples and associated data were obtained from routine diagnostic activities at the Montpellier University Hospital and were included in the institutional biobank (DIRTEC‐MI; AC‐2024‐6656), in accordance with French regulatory requirements.

### 2.2 HCV RNA Detection and Quantification

Plasma and DBS specimens were tested using the Alinity m HCV assay (Abbott Molecular). The quantification range of the assay extends from 12 to 1 × 10^8^ IU/mL (1.1–8.0 log_10_ IU/mL), with a limit of detection (LOD) of 5.11 IU/mL in plasma, according to the manufacturer.

Capillary blood was absorbed onto a Protein Saver 903 Card (Whatman, Dassel, Germany) to completely fill 12‐mm preprinted circular paper disks. Cards were sent at ambient temperature by mail in zipped plastic bags. Upon arrival, DBS were stored at −20°C for 1 to 8 days until analysis. Four 6‐mm punches were eluted in 500 μL of PBS–BSA (10%)/Tween‐20 (0.05%). After 1 h of incubation, the DBS eluate was centrifuged, the supernatant was collected and diluted with PBS to reach a final volume of 1300 μL for automated extraction and RT‐PCR using the Alinity m platform.

### 2.3 Analytical Validation and Comparative Quantification of HCV RNA in DBS

The LOD for HCV RNA on DBS was determined using twofold serial dilutions of HCV‐spiked whole blood. Each dilution was tested 10 times and results were complemented with data obtained from the comparative plasma–DBS analysis. Repeatability (intra‐run variability) and reproducibility (inter‐run variability) were evaluated at two predefined viral load levels (100 IU/mL and 10 000 IU/mL of DBS eluates). Each condition was tested in 10 replicates for repeatability and in 10 replicates across separate days and analytical runs for reproducibility.

We conducted a comparative analysis of HCV RNA quantification in venous plasma and DBS samples with moderate to low viral loads. Twenty EDTA‐anticoagulated blood samples were selected. Each sample was initially diluted 1:20 in HCV‐, HBV‐ and HIV‐free EDTA whole blood, subsequently serially diluted in twofold steps to obtain the following final dilutions: 1:20, 1:160, 1:640, 1:1280, 1:2560 and 1:5120. For each dilution, DBS were prepared by spotting 50 μL of diluted whole blood onto filter paper cards. Cards were dried overnight at room temperature, stored at −20°C and equilibrated at room temperature before punching. A total of 114 DBS prepared from diluted venous blood were analysed and HCV RNA quantification from DBS punches was compared with the corresponding plasma HCV RNA values.

### 2.4 Plasma Viral Load Distribution at Diagnosis as Reference for DBS Analytical Performance

To characterise the distribution of viral loads in untreated individuals, we retrospectively analysed HCV RNA quantification results obtained from venous blood samples collected at Montpellier University Hospital between January 2020 and December 2024. The resulting proportion was then extrapolated to the population screened using DBS. This analysis enabled estimation of the potential risk of false‐negative results associated with the analytical sensitivity limits of DBS, based on the proportion of cases with low‐level viremia under real‐life screening conditions. Confidence intervals (95% CI) for proportions were calculated using the Wilson score method. Only the first sample per patient was included and viral load measurements were obtained from standard venous plasma samples.

### 2.5 Statistical Analysis

Repeatability and reproducibility were evaluated by calculating the mean, standard deviation (SD) and coefficient of variation (CV) of HCV RNA measurements obtained across intra‐assay and inter‐assay runs at predefined low and high viral load levels. The LOD was determined using probit regression analysis to identify the concentrations at which 95% of replicates yielded a detectable or quantifiable result, respectively. The functional limit of quantification (LOQ) on DBS was estimated using probit regression and defined as the concentration in eluate for which ≥ 95% of replicates yielded quantifiable results (reported value ≥ 12 IU/mL, corresponding to the manufacturer's LOQ). Correlation between HCV RNA levels measured in DBS and plasma samples was assessed using Deming regression analysis. Agreement between paired measurements was evaluated using Bland–Altman plots, with calculation of the mean bias and 95% limits of agreement. The resulting proportion was then extrapolated to the population screened using DBS. Confidence intervals (95% CI) for proportions were calculated using the Wilson score method.

Statistical analyses were conducted using GraphPad Prism version 9.0.0 (GraphPad Software, La Jolla, CA, USA) and R version 2024.12.1 + 563 (R Foundation for Statistical Computing, Vienna, Austria). A *p* < 0.05 was considered statistically significant. We used the SageR and ggeffects packages. Deming regression analysis and graphical outputs were generated using the Deming and ggplot2 packages, respectively.

## 3 Results

### 3.1 Analytical Performance and Detection Limits of HCV RNA Quantification on DBS

**FIGURE 1:**
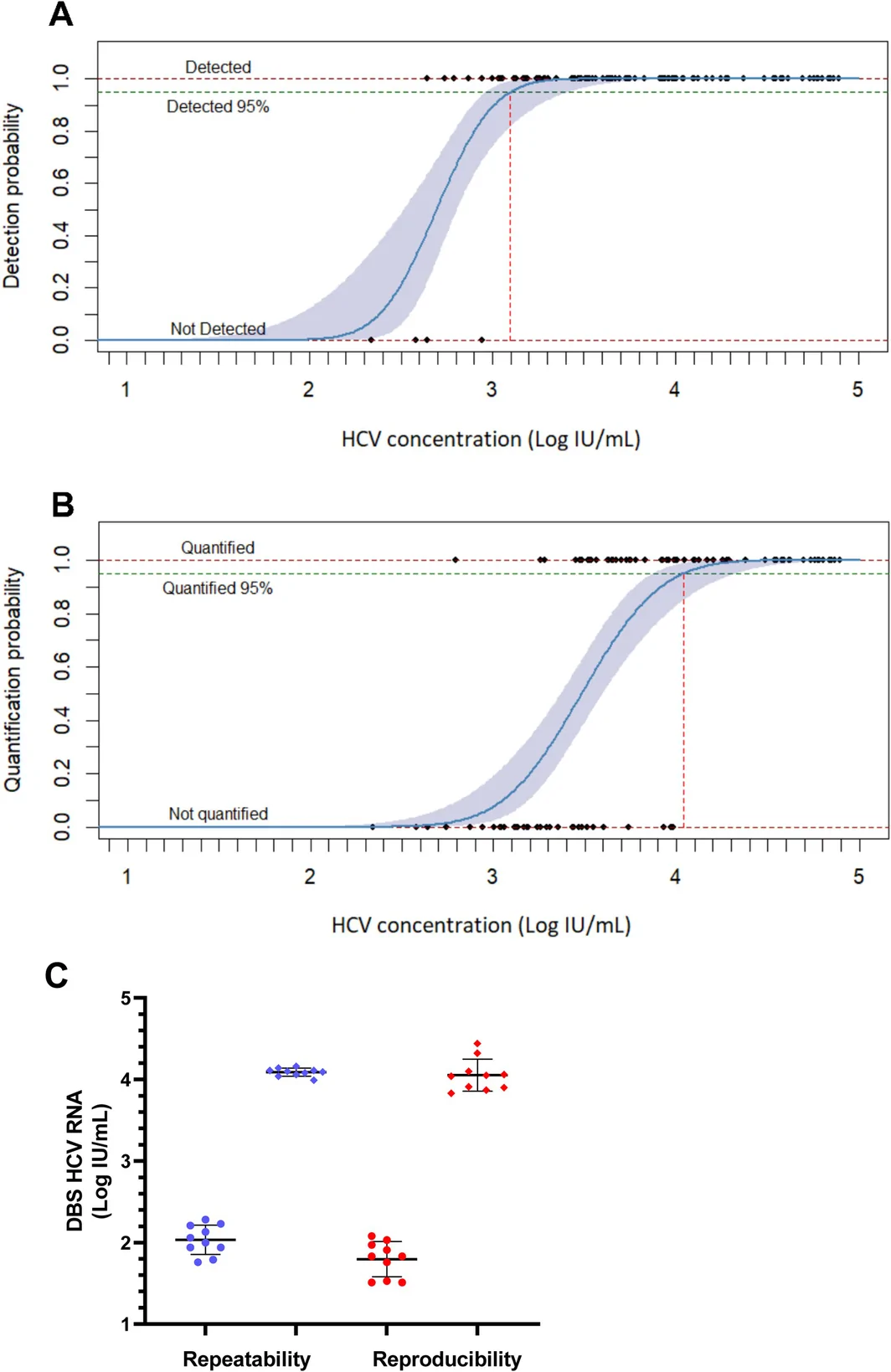
Analytical performance of HCV RNA detection and quantification from DBS samples. The probability of HCV RNA detection (A) and quantification (B) was modelled using probit regression based on 1:2 serial dilutions of HCV‐spiked whole blood spotted onto DBS cards. The limit of detection (LOD) and limit of quantification (LOQ) were defined as the plasma HCV RNA concentrations at which 95% of replicates were detected or quantified, respectively, using the DBS protocol. These thresholds were estimated at 1252 IU/mL (3.09 log_10_ IU/mL) for the LOD and 10 877 IU/mL (4.04 log_10_ IU/mL) for the LOQ. Blue curves represent the modelled detection or quantification probability as a function of log‐transformed HCV RNA concentration, with individual results shown as detected/quantified or not detected/unquantified. Shaded blue areas indicate the 95% confidence intervals. Horizontal green dotted lines indicate the 95% probability threshold and vertical red dotted lines indicate the corresponding LOD or LOQ values. (C) Repeatability and reproducibility of HCV RNA quantification on DBS. Analytical precision was assessed at two HCV RNA concentrations in DBS eluates: a low level (~2 log_10_ IU/mL, blue) and a high level (~4 log_10_ IU/mL, red). Repeatability (intra‐assay) and reproducibility (inter‐assay) were evaluated using 10 replicates per level. Mean ± SD values were 2.034 ± 0.181 and 1.796 ± 0.215 log_10_ IU/mL at the low level and 4.088 ± 0.049 and 4.052 ± 0.197 log_10_ IU/mL at the high level, for repeatability and reproducibility, respectively.

Analytical performance of DBS‐based HCV RNA testing, including sensitivity and precision, is summarized in Figure 1. The analytical sensitivity of HCV RNA quantification on DBS showed a limit of detection (LOD) of 1252 IU/mL (95% CI: 824–1902), corresponding to a 95% detection rate in dilution experiments. An approximate LOQ on DBS was estimated at 10877 IU/mL (95% CI: 6753–17 517), corresponding to the plasma concentration at which ≥ 95% of DBS replicates yielded quantifiable HCV RNA levels (≥ 12 IU/mL in eluate).

**FIGURE 2:**
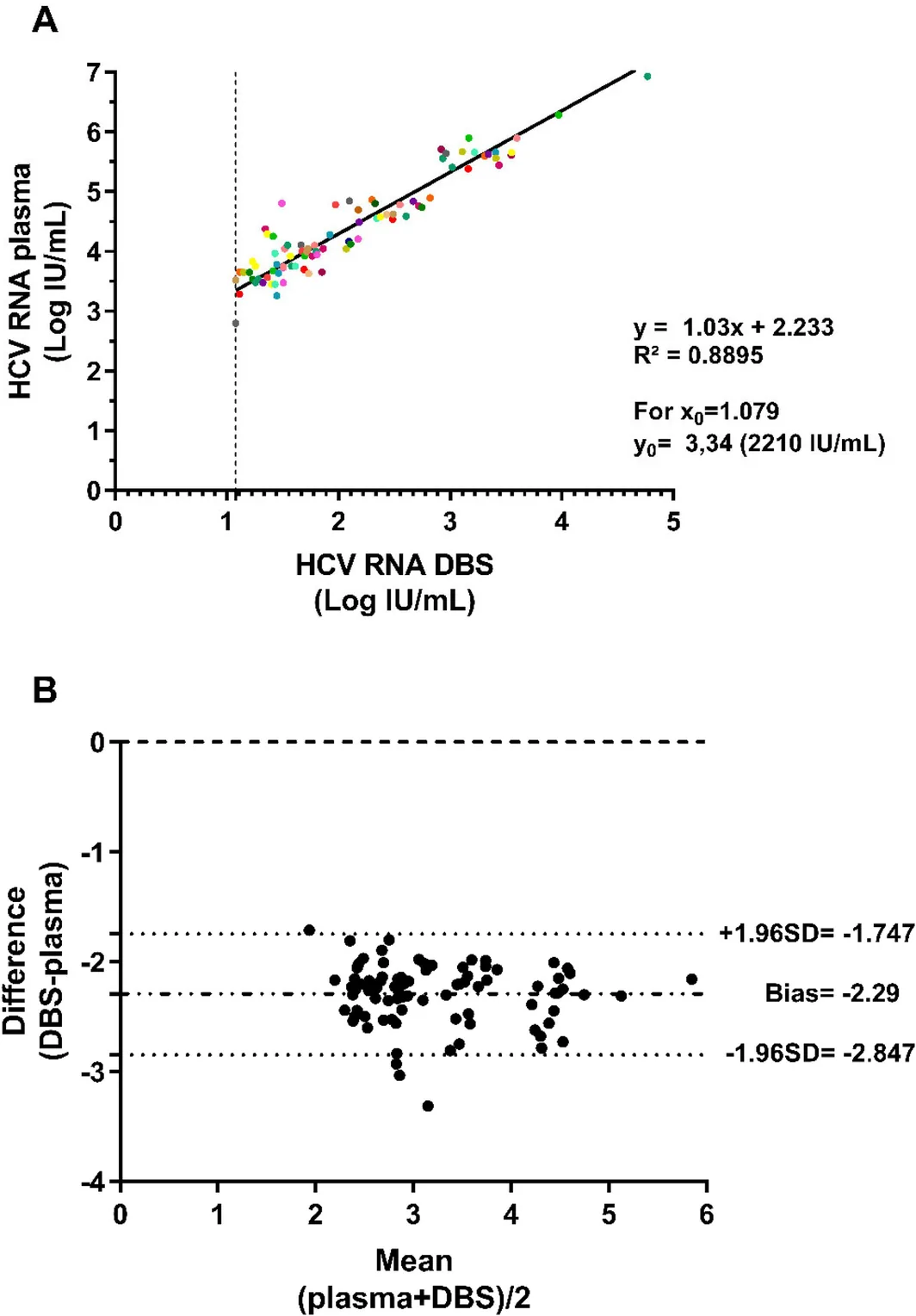
Correlation and agreement between HCV RNA quantification in DBS and plasma. (A) Linear regression analysis comparing HCV RNA levels measured in DBS and matched plasma samples prepared by serial dilution of 19 HCV RNA positive specimens (*n* = 113). The vertical dashed line indicates the lower limit of quantification in DBS eluate (12 IU/mL, 1.07 log IU/mL), corresponding to a theoretical plasma concentration of 3.34 log_10_ IU/mL (2210 IU/mL) according to the regression model. (B) Bland–Altman plot showing the difference in HCV RNA levels between DBS and plasma as a function of the mean of both measurements. The mean bias was −2.29 log IU/mL, with 95% limits of agreement ranging from −2.84 to −1.74 log IU/mL.

A strong correlation was observed between DBS and plasma HCV RNA measurements (Figure 2A; R^2^ = 0.88, *p* < 0.0001). Bland–Altman analysis demonstrated a mean bias of −2.29 log_10_ IU/mL, indicating a systematic underestimation of HCV RNA levels in DBS compared with plasma, with narrow limits of agreement across the concentration range (Figure 2B). Based on the regression model, the theoretical plasma viral load corresponding to the DBS LOD was estimated at 2210 IU/mL, showing reasonable agreement with the experimentally determined LOD. Together, these findings confirm the internal consistency of the analytical performance estimates, while highlighting the expected risk of false‐negative results at low‐level viremia.

### 3.2 Results and Coverage of HCV Screening Through DBS Between 2020 and 2024

**FIGURE 3:**
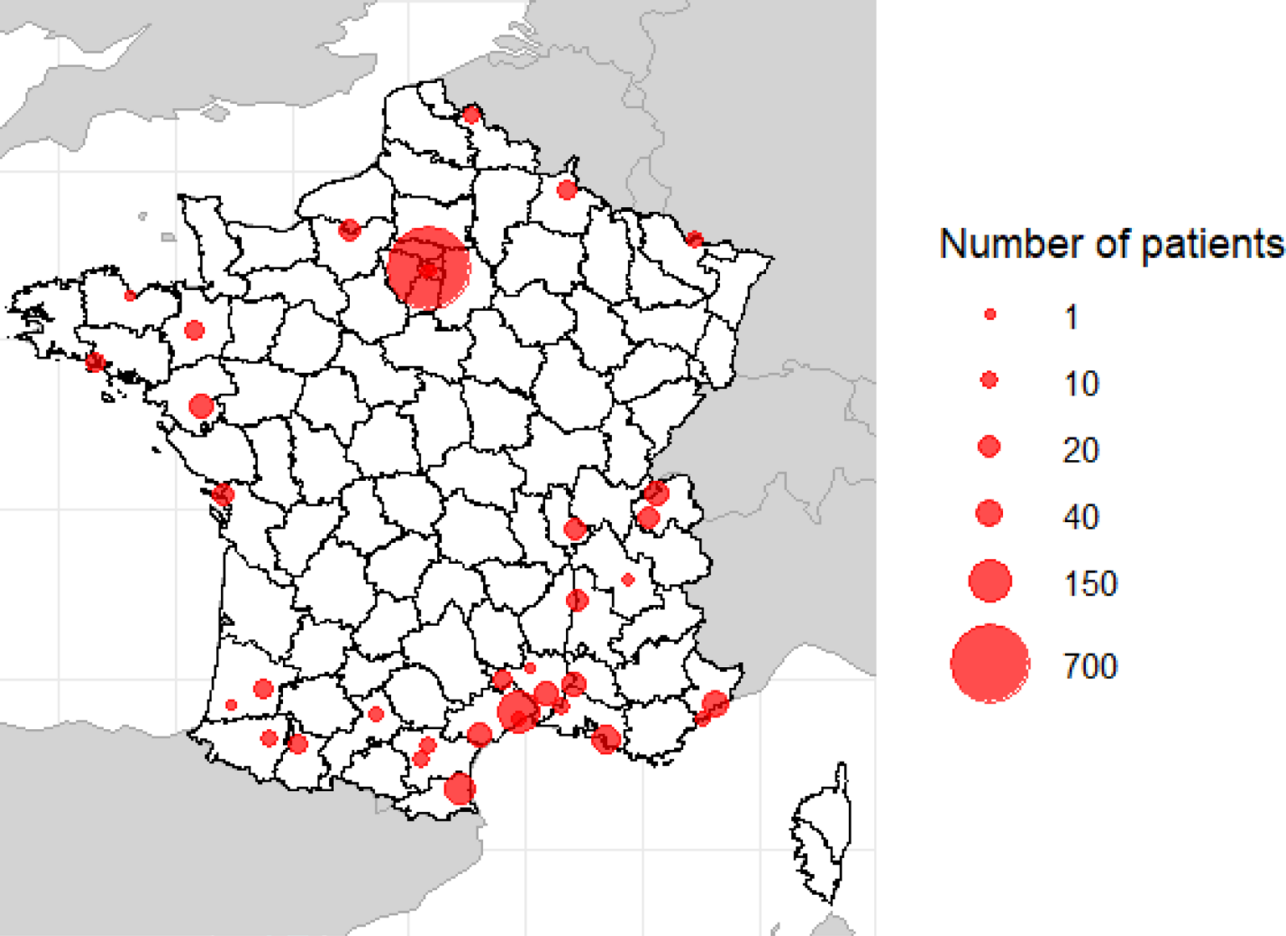
Geographical distribution of DBS samples submitted for HCV RNA testing (2020–2024). Map showing the locations and relative numbers of individuals from harm reduction centres tested for HCV RNA on DBS between 2020 and 2024. The size and colour intensity of each point represent the number of individuals tested per location.

Between 2020 and 2024, a total of 3518 DBS samples were received and analysed for HCV testing. Samples originated from multiple regions across France, with a strong representation from southern France and the Paris area (Figure 3). The median age was 43 years (IQR: 37–50) and the male‐to‐female sex ratio was 4.2 (data not shown).

A total of 1552 individuals were tested for HCV RNA; 1325 were HCV antibody–positive (37.6%), while 227 were HCV antibody–negative and underwent HCV RNA testing following a specific request by the prescribing centre, irrespective of the serological screening result (Table 1). Overall, 428 individuals (27.6%; 95% CI: 25.4–29.9) were HCV RNA positive (Table 1). HCV RNA was detected in 423 of 1325 (31.9%) HCV antibody–positive individuals (95% CI: 29.5–34.5). HCV RNA was also detected in 5 of 227 (2.2%) individuals testing negative for HCV antibodies (95% CI: 0.9–5.1). Of the HCV RNA–positive individuals, 89.8% had quantifiable HCV RNA levels, while 10.2% exhibited detectable but non‐quantifiable HCV RNA.

**TABLE 1:** Result of HCV RNA detection among patients according to HCV serological status on corresponding DBS.

Group	Individuals, *n* (%)	HCV RNA positive (%)	95% CI
Anti‐HCV positive	1325 (85.4%)	423 (31.9%)	29.5–34.5
Anti‐HCV negative	227 (14.6%)	5 (2.2%)	0.9–5.1
Total	1552 (100%)	428 (27.6%)	25.4–29.9

### 3.3 Frequency of Low‐Level HCV RNA and Extrapolation of False‐Negative Molecular Results in DBS Screening

We estimated the number of missed HCV diagnoses due to low‐level viremia based on the distribution of plasma viral loads observed in our centre. Among 331 HCV‐infected, untreated individuals who tested positive for HCV RNA at Montpellier University Hospital between 2020 and 2024, plasma HCV RNA levels were below the DBS limit of detection (< 1252 IU/mL) in nine cases (2.72%; 95% CI: 1.44%–5.09%). Applying this proportion to the 428 HCV RNA‐positive individuals identified through DBS screening suggests that approximately 12 additional viremic infections may have remained undetected because of low HCV RNA levels below the DBS detection threshold.

## 4 Discussion

**FIGURE 4:**
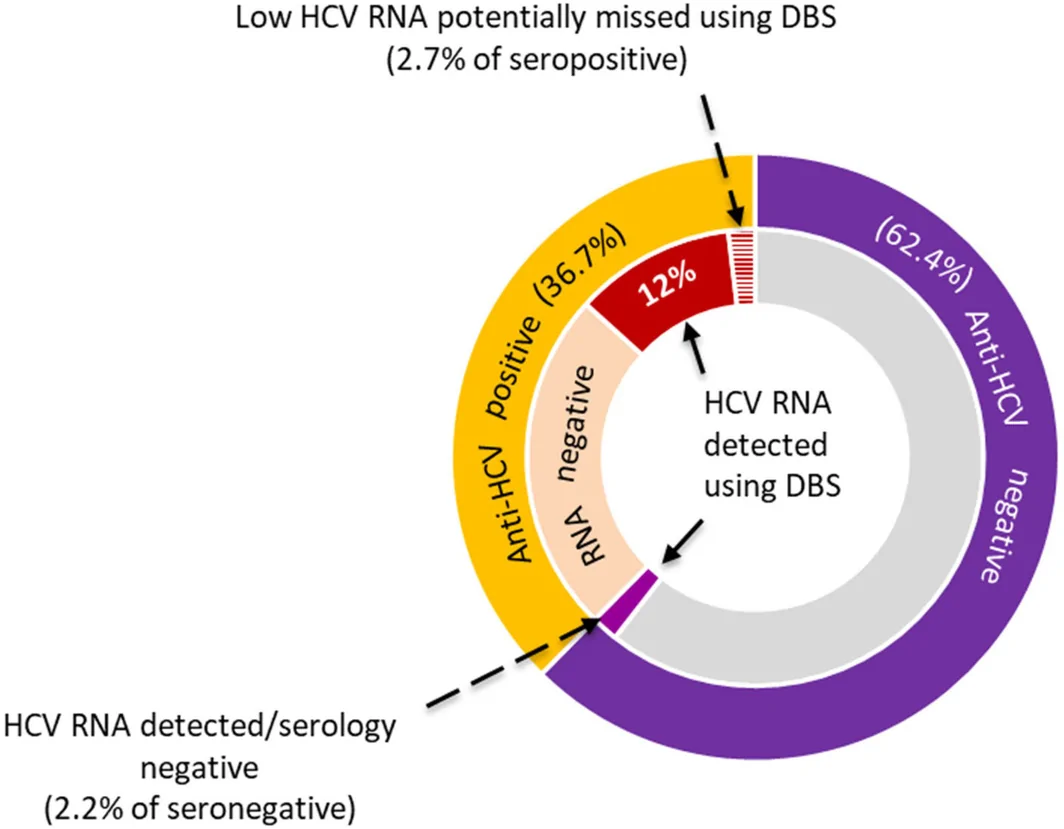
Overview of DBS‐based HCV RNA testing with potential missed infections and opportunities for early diagnosis. Schematic representation of HCV serological status and HCV RNA detection outcomes using DBS in a highly exposed population. The outer ring shows the distribution of anti‐HCV serological status, with 37.6% anti‐HCV–positive and 62.4% anti‐HCV–negative individuals. The inner ring represents HCV RNA detection using DBS, with an overall HCV RNA detection rate of 12%. Among anti‐HCV–positive individuals, a small proportion with low‐level viremia may remain undetected due to the analytical sensitivity limits of DBS PCR. In addition, a small proportion of anti‐HCV–negative individuals may harbour acute HCV infection with detectable HCV RNA, potentially missed by serological screening alone.

Achieving HCV elimination critically depends on the ability to reach populations who remain largely excluded from conventional healthcare pathways. In this context, we report a 5‐year real‐life evaluation of DBS–based HCV screening implemented in community settings targeting populations at increased risk of infection. Since 2019, France has publicly committed to eliminating HCV by 2025, aiming to diagnose 90% of people living with the virus and treat 80% of them. This objective has been supported by the removal of access restrictions to DAAs since 2016 and by the gradual decentralisation of DAA prescribing and dispensing through general practitioners and pharmacies. However, recent European monitoring data suggest that achieving these elimination targets remains uncertain, as substantial gaps persist in HCV testing coverage, linkage to care and treatment uptake among key populations [16]. Our results indicate that DBS‐based approaches can have a substantial clinical impact by extending access to HCV diagnosis among underserved populations. Figure 4 summarises both the opportunities for HCV diagnosis and the risk of missed infections associated with DBS‐based HCV RNA screening strategies.

When implemented in high‐prevalence, community‐based settings, DBS‐based screening yields a high diagnostic return. The geographical spread and number of DBS samples tested reflect the method's acceptability and successful integration into routine practice for populations at risk of HCV. Notably, over the study period, our laboratory identified more HCV RNA–positive individuals through DBS testing than through conventional blood‐based sampling collected in our hospital. In a previous study conducted between 2018 and 2023 in 26 community‐based addiction care facilities, the French National Reference Centre for HCV reported the presence of anti‐HCV antibodies in 143 out of 587 individuals (24.4%) and HCV RNA detection in 96 of them (67.1%) [17]. Among the 96 newly diagnosed patients, 80 received treatment with DAAs and the virological cure rate was 90.0% [17]. Widespread use of DBS is feasible; as early as 2013, DBS sampling accounted for more than 20% of new HCV diagnoses in addiction treatment services across Scotland [18]. In contrast to targeted screening strategies, HCV testing in the general population is characterised by a markedly lower diagnostic yield. During a universal HCV screening campaign conducted in Montpellier in 2019, free venous blood testing was offered across public and private laboratories [19]. Among 10 143 individuals screened, only 91 (0.89%) tested positive for anti‐HCV antibodies and nine were HCV RNA–positive, corresponding to a positivity rate of 0.09% [19].

The clinical value of a diagnostic strategy depends on the intrinsic analytical performance of the assay and its practical accessibility in real‐world conditions. Optimal outcomes are achieved when testing strategies reach the highest proportion of infected individuals and correctly identify those with active infection. DBS sampling is a method well‐suited to decentralized screening practices, alongside rapid serological tests and point‐of‐care molecular tests on capillary blood [20, 21]. Centralised testing of DBS samples collected outside laboratory settings avoids the need to deploy multiple molecular testing devices and the constraints associated with operating them in non‐laboratory environments.

To better interpret the clinical implications of DBS‐based testing, we specifically assessed the analytical sensitivity of HCV RNA detection from DBS samples. The LOD for HCV RNA quantification from DBS was established at 1252 IU/mL, based on the concentration at which 95% of replicates yielded a detectable signal across multiple analytical runs. Using a second approach based on the plasma–DBS correlation curve, the plasma‐equivalent LOD was estimated at 2210 IU/mL. Using the same platform, reliable detection of DBS‐based HCV RNA has previously been reported at lower plasma viral loads, around 500 IU/mL [22]. In this study, analytical sensitivity was inferred from serial dilution experiments rather than from formal probit analysis and DBS elution was performed using a proprietary manufacturer‐provided buffer, which may have contributed to optimised RNA recovery.

To place these analytical performance data from a clinical perspective, we next evaluated the proportion of HCV‐infected individuals who might remain undetected due to very low‐level viremia. We observed that less than 3% of untreated individuals receiving care in our hospital had plasma HCV RNA concentrations falling below the LOD on DBS (< 1252 IU/mL). Our observation is consistent with previously reported data on low‐level viremia in treatment‐naïve individuals. The median HCV viral load among treatment‐naïve individuals has been reported at approximately 2.5 × 10^5^ IU/mL, with an interquartile range from 1.0 × 10^5^ to 6.3 × 10^5^ IU/mL [23], confirming that most untreated patients exhibit high levels of viremia. In a Swiss study including 2533 treatment‐naïve persons, 133 (5.3%) had a viral load below 3000 IU/mL and among these, 88 (3.5%) had levels below 1000 IU/mL [24]. Similarly, Fytili et al. reported that 82 (7.1%) of 1775 HCV‐infected individuals had viral loads below 600 IU/mL, including 77 who were untreated, further supporting the observation that low‐level viremia occurs in a small subset of patients [25]. A large global analysis of 66 640 individuals showed that an HCV RNA detection threshold around 1300 IU/mL would still identify approximately 97% of viraemic infections, suggesting that extremely low analytical limits of detection may not be necessary to maintain high clinical sensitivity [26]. Low‐level viraemia has been associated with higher proportions of spontaneous viral clearance [27, 28]. Finally, our estimate that less than 3% of infections may be undetected aligns with findings from the meta‐analysis used to inform the WHO recommendations, which reported pooled sensitivity and specificity values of 98% (95% CI: 95–99) [7]. Several pre‐analytical factors may influence DBS assay performance, including blood spot quality, blood volume, drying procedures, storage conditions and transport. Previous studies have highlighted the impact of these variables on analyte stability, analytical performance, assay sensitivity and the proportion of samples deemed unsuitable for analysis [29, 30, 31].

Beyond analytical sensitivity limitations, sequential testing strategies in which molecular testing follows serological screening may contribute to missed diagnoses. The detection of HCV RNA in anti‐HCV–negative individuals underscores the value of direct RNA testing among populations at high risk of infection [32]. These cases likely reflect acute or very recent infections occurring during the serological window period. Our observation is consistent with European surveillance data indicating that acute infections account for approximately 5% of reported HCV cases and are predominantly observed among PWID [33]. Therefore, a strategy relying on systematic molecular DBS‐based assays among individuals at high risk of acute HCV infection could compensate, at least in part, for cases missed due to lower analytical sensitivity.

Despite increasing evidence supporting DBS‐based molecular testing, regulatory constraints remain a major barrier to large‐scale implementation. DBS‐based viral load testing has previously been approved for HIV RNA quantification on the Abbott architect platform [34]; however, the use of DBS with the Alinity HCV assay relies on an off‐label protocol, as no regulatory approval has yet been granted for this platform. In 2017, the WHO highlighted this gap as an implementation limitation, despite recommending DBS to improve access to hepatitis testing [35]. Eight years later, it appears that major manufacturers of molecular diagnostic assays have not pursued regulatory approval for HCV RNA testing, nor included DBS in the intended use of their assays. This persistent regulatory limitation continues to hinder the large‐scale implementation of DBS‐based molecular HCV screening.

A limitation of our analysis is that the estimation of missed diagnoses assumes a similar viral load distribution between untreated patients diagnosed in hospital settings and individuals screened through community‐based DBS programmes. Because detailed clinical information, including injection drug use status, reinfection history and acute versus chronic infection status, was not systematically available for DBS‐screened individuals, this assumption could not be formally verified. However, given the consistently low prevalence of very low‐level viremia reported across different untreated populations, we believe that the resulting estimate provides a reasonable approximation of the potential impact of DBS analytical sensitivity on case detection. Future studies could extend this approach by evaluating the overall diagnostic yield of DBS‐based screening strategies, integrating both analytical performance and accessibility to care in high‐risk populations.

Our evaluation demonstrates that DBS‐based HCV screening is a robust and operationally effective strategy for expanding access to virological diagnosis in underserved populations. Despite a modest reduction in analytical sensitivity compared with plasma‐based testing, DBS testing enabled the identification of a substantial number of HCV RNA–positive individuals who would likely not have been tested using conventional venous blood sampling. The small proportion of infections potentially missed due to very low‐level viremia appears limited and is outweighed by the significant public health benefit conferred by increased testing coverage and improved reach to high‐risk populations. In the context of HCV elimination efforts, DBS‐based molecular screening represents a pragmatic and scalable tool that can complement existing testing strategies, provided that regulatory barriers are addressed to support its broader implementation.

## Funding

The FHU TIE (University Hospital Federation ‘Triangular analysis of interactions between host, infectious agent and environment’) and the Montpellier University Hospital.

## Conflicts of Interest

Edouard Tuaillon has received honoraria from Gilead Sciences. All remaining authors have no potential conflicts to declare.

## Data Availability

The data that support the findings of this study are available from the corresponding author upon reasonable request.
